# A Glucuronoxylomannan-Associated Immune Signature, Characterized by Monocyte Deactivation and an Increased Interleukin 10 Level, Is a Predictor of Death in Cryptococcal Meningitis

**DOI:** 10.1093/infdis/jiw007

**Published:** 2016-01-14

**Authors:** James E. Scriven, Lisa M. Graham, Charlotte Schutz, Thomas J. Scriba, Katalin A. Wilkinson, Robert J. Wilkinson, David R. Boulware, Britta C. Urban, David G. Lalloo, Graeme Meintjes

**Affiliations:** 1 Liverpool School of Tropical Medicine; 2 Wellcome Trust Liverpool Glasgow Centre for Global Health Research, Liverpool; 3 Department of Medicine, Imperial CollegeLondon; 4 Mill Hill Laboratory, Francis Crick Institute,London, United Kingdom; 5 Clinical Infectious Diseases Research Initiative; 6 South African TB Vaccine Initiative, Institute of Infectious Disease and Molecular Medicine; 7 Department of Paediatrics and Child Health, University of Cape Town; 8 Department of Medicine, University of Cape Town and Groote Schuur Hospital, South Africa; 9 Department of Medicine, University of Minnesota, Minneapolis

**Keywords:** *Cryptococcus neoformans*, cryptococcal meningitis, HIV, mortality, monocytes, HLA-DR, TNF-alpha, IL-10, flow cytometry, principal component analysis

## Abstract

***Background.*** Cryptococcal meningitis remains a significant cause of death among human immunodeficiency virus type 1 (HIV)–infected persons in Africa. We aimed to better understand the pathogenesis and identify immune correlates of mortality, particularly the role of monocyte activation.

***Methods.*** A prospective cohort study was conducted in Cape Town, South Africa. Patients with a first episode of cryptococcal meningitis were enrolled, and their immune responses were assessed in unstimulated and stimulated blood specimens, using flow cytometry and cytokine analysis.

***Results.*** Sixty participants were enrolled (median CD4^+^ T-cell count, 34 cells/µL). Mortality was 23% (14 of 60 participants) at 14 days and 39% (22 of 57) at 12 weeks. Nonsurvivors were more likely to have an altered consciousness and higher cerebrospinal fluid fungal burden at presentation. Principal component analysis identified an immune signature associated with early mortality, characterized by monocyte deactivation (reduced HLA-DR expression and tumor necrosis factor α response to lipopolysaccharide); increased serum interleukin 6, CXCL10, and interleukin 10 levels; increased neutrophil counts; and decreased T-helper cell type 1 responses. This immune signature remained an independent predictor of early mortality after adjustment for consciousness level and fungal burden and was associated with higher serum titers of cryptococcal glucuronoxylomannan.

***Conclusions.*** Cryptococcal-related mortality is associated with monocyte deactivation and an antiinflammatory blood immune signature, possibly due to *Cryptococcus* modulation of the host immune response. Validation in other cohorts is required.

Cryptococcal meningitis, caused by the encapsulated yeast *Cryptococcus neoformans*, is the commonest form of adult meningitis in sub-Saharan Africa and remains a significant cause of death among human immunodeficiency virus type 1 (HIV)–infected individuals in this region [1–3]. Despite availability of amphotericin B and antiretroviral therapy (ART), mortality remains high, ranging between 17% and 32% at 14 days and between 34% and 59% at 12 weeks [4, 5]. Reduced consciousness level, high fungal burden, and slower yeast clearance from the cerebrospinal fluid (CSF) have been identified as independent predictors for mortality [4]. However, some patients with a normal consciousness level at the time of diagnosis may deteriorate and die despite optimal treatment. Secondary infections such as bacterial sepsis or tuberculosis can also contribute [4, 6].

The host immune response is central to the pathogenesis of cryptococcosis. The vast majority of cryptococcal infections occur among individuals with impaired cell-mediated immunity, and the marked susceptibility of persons with AIDS demonstrates the importance of CD4^+^ T lymphocytes in protection [7]. However, low CD4^+^ T-cell counts have not consistently been identified as a risk factor for mortality among persons with AIDS-related cryptococcosis, nor has the number of circulating *Cryptococcus*-specific CD4^+^ T lymphocytes [4, 8]. Animal models of cryptococcosis demonstrate the microbiological and survival benefits of a T-helper type 1 (Th1) response, characterized by interferon γ (IFN-γ) production and classical macrophage activation, along with the detrimental effects of a Th2 response, characterized by interleukin 4 (IL-4) and interleukin 13 (IL-13) production with alternative macrophage activation [9–11]. Studies in humans have not demonstrated any association between a Th2 response and poor outcome but have observed a significantly higher proportion of circulating cryptococcal-specific CD4^+^ T cells that produce both IFN-γ and tumor necrosis factor α (TNF-α) in survivors [8]. This is consistent with previous studies that have observed higher CSF concentrations of such cytokines in survivors and significantly improved CSF fungal clearance when adjunctive IFN-γ was given alongside antifungal therapy [12, 13]. Despite their importance in the murine immune response, the relationship between the activation state of macrophages and their blood precursor monocytes with clinical outcome has not been studied in persons with HIV-associated cryptococcosis. To address this, we examined immune responses in blood, using ex vivo stimulation assays, flow cytometry, and cytokine analysis. We hypothesized that a less activated monocyte phenotype would be associated with mortality.

## METHODS

### Study Design

A prospective cohort study was conducted between April 2012 and January 2014 at GF Jooste, Khayelitsha, and Mitchell's Plain Hospitals, Cape Town, South Africa. Consecutive patients aged ≥18 years with a first episode of HIV-associated cryptococcal meningitis were enrolled within 48 hours of starting antifungal therapy. At enrollment, CSF samples were collected and quantitative culture performed to quantify fungal burden, as previously described [14]. A peripheral blood specimen was obtained to assess the immune response. Antifungal therapy comprised intravenous amphotericin B (1 mg/kg/day) and oral fluconazole 800 mg/day for 14 days, followed by fluconazole 400 mg/day for a further 10 weeks and then maintenance fluconazole. Additional lumbar punctures with therapeutic CSF drainage were performed at attending clinicians' discretion to control raised intracranial pressure. ART was initiated 4 weeks after meningitis diagnosis according to national guidelines, unless patients were receiving it at enrollment [15]. The primary end point was all-cause mortality at 14 days; the secondary end point was mortality at 12 weeks. All participants provided written informed consent; surrogate consent was sought from the next of kin for patients with impaired consciousness. Ethical approval was obtained from the research ethics committees of the University of Cape Town (reference numbers 408/2010 and 371/2013) and Liverpool School of Tropical Medicine (reference number 11.92).

### Whole-Blood Flow Cytometry

**Figure 1.**
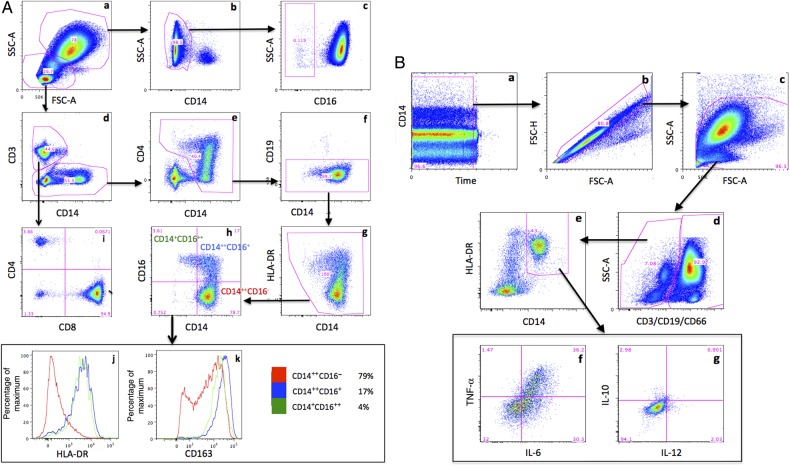
Flow cytometry gating strategy (representative plots). *A*, Circulating cell populations (unstimulated blood). *a*, Following exclusion of doublets and antibody aggregates, cells were separated into neutrophils and nonneutrophils by physical characteristics. *b*, CD14^+^ monocytes removed from neutrophils population. *c*, CD16^−^ (apoptotic) neutrophils identified. *d*, nonneutrophils split into T cells (CD3^+^) and non–T cells (CD3^−^). *e*, Monocytes identified from non–T cells, using CD14^+^. *f*, CD19 used to exclude B cells from monocyte population. *g*, Removal of CD14^−^ and HLA-DR^−^ cells from monocyte population. *h*, Monocytes split into classical (CD14^++^CD16^−^; red), intermediate (CD14^++^CD16^+^; blue), and nonclassical (CD14^+^CD16^++^; green) subtypes. *i*, CD4^+^, CD8^+^, and CD4^−^CD8^−^ T cells identified. *j*, Histogram showing expression of HLA-DR on monocyte subsets. *k*, Histogram showing expression of CD163 on monocyte subsets. FSC-A, forward scatter, area; SSC-A, side scatter, area. All axes apart from FSC-A, SSC-A, and the percentage of maximum are log_10_ scale. *B*, Monocyte cytokine production (lipopolysaccharide-stimulated blood). *a*, Cellular debris from end of acquisition removed. *b*, Doublets removed. *c*, Debris removed. *d*, Dump channel used to remove T cells, B cells, and neutrophils. *e*, Monocytes identified using CD14 and HLA-DR. *f*, Expression of interleukin 6 (IL-6) and tumor necrosis factor α (TNF-α). *g*, Expression of interleukin 12 (IL-12) and interleukin 10 (IL-10). Positive gates in panels *f* and *g* are drawn using the unstimulated sample. This figure is available in black and white in print and in color online.

Flow cytometry was performed on unstimulated blood specimens from 56 participants. Fresh whole-blood specimens were stained with commercially available conjugated monoclonal antibodies to assess phenotype and activation of both T-cell and monocyte subsets. Variables measured included the relative frequency of circulating neutrophils, monocytes, CD4^+^ T cells, CD8^+^ T cells, and CD4^−^CD8^−^ T cells (recorded as the percentage of white blood cells [WBCs]); the proportion of CD16^−^ neutrophils (recorded as the percentage of total neutrophils); the proportion of classical (CD14^++^CD16^−^), intermediate (CD14^++^, CD16^+^), and nonclassical (CD14^+^CD16^++^) monocytes; the expression of HLA-DR, CD38, and PD-1 on CD4^+^, CD8^+^, and CD4^−^CD8^−^ T cells; and the expression of HLA-DR, CD163, PD-1, CCR2, CCR5, CD80, and Toll-like receptor 4 (TLR4) on monocytes (determined for the whole population and for the 3 monocyte subsets described above). Further details of the antibodies used are given in the Supplementary Methods; the gating strategy is illustrated in Figure 1*A*.

### Antigen-Stimulation Assay

Fresh whole-blood specimens were stimulated for 6 and 24 hours at 37°C with lipopolysaccharide (LPS; a TLR4 agonist; Invivogen, San Diego, California); the imidazoquinoline compound R848 (a TLR7/8 agonist; Invivogen); *Cryptococcus* capsule glucuronoxylomannan (GXM); heat-killed, mechanically disrupted *C. neoformans* H99; or an equivalent volume of phosphate-buffered saline (unstimulated). Supernatants from whole-blood specimens stimulated for 24 hours were removed and stored at −80°C for later cytokine analysis (see below). Cytokine production was assessed in whole-blood specimens stimulated for 6 hours, using intracellular cytokine staining. Variables measured for each of the 4 stimulations included relative frequency of neutrophils and monocytes (recorded as the percentage of WBCs); neutrophil expression of CD66 (recorded as the mean fluorescence intensity [MFI]) and CD16 (recorded as the percentage of cells); and expression of HLA-DR (recorded as the MFI) and production of interleukin 6 (IL-6), interleukin 10 (IL-10), interleukin 12 (IL-12), and TNF-α by circulating monocytes (recorded as the percentage of monocytes positive for cytokine). HLA-DR expression was recorded as absolute MFI value and relative expression compared to unstimulated. Results from antigen-stimulation assays were available for 41–48 participants (depending on stimulation). Owing to autofluorescence of the cryptococcal preparations, IL-12 and IL-10 expression could not be measured accurately for cryptococcal stimulations. Further details regarding the preparation of GXM and the antibodies and fluorochromes used are given in the Supplementary Methods; the gating strategy is illustrated in Figure 1*B*.

### Biomarker Analysis

The concentrations of 26 cytokines/chemokines were measured in serum specimens and supernatant of blood specimens subjected to antigen stimulation for 24 hours, using a Luminex multiplex platform (Invitrogen; Carlsbad, California). These comprised interleukin 1 receptor antagonist (IL-1RA), IL-1β, interleukin 2 (IL-2), IL-2R, IL-4, interleukin 5 (IL-5), IL-6, interleukin 7, interleukin 8 (IL-8), IL-10, IL-12p40/p70, IL-13, interleukin 15, interleukin 17 (IL-17), granulocyte colony–stimulating factor, granulocyte-macrophage colony-stimulating factor (GM-CSF), TNF-α, IFN-α, IFN-γ, vascular endothelium growth factor, C-C motif chemokine ligand 2 (CCL2), CCL3, CCL4, CCL5, C-X-C motif chemokine ligand 9 (CXCL9), and CXCL10. Eight biomarkers were consistently undetectable in unstimulated serum specimens and excluded from that analysis (IL-2, IL-4, IL-5, IL-13, IL-17, GM-CSF, IFN-γ, and TNF-α); all biomarkers were detectable in stimulated blood specimens. The concentrations of 2 soluble markers of macrophage activation (soluble CD163 [sCD163] and sCD14) were measured in unstimulated serum, using an enzyme-linked immunosorbent assay (R&D, Minneapolis, Minnesota). A semiquantitative measurement of serum GXM titer was made by performing 2-fold serial dilutions and testing each dilution with a cryptococcal antigen lateral flow assay (Immy, Norman, Oklahoma). Routine hematological and biochemical tests were performed by the National Health Laboratory Service at Groote Schuur Hospital, Cape Town. Biomarker concentrations below the detectable limit for the assay were ascribed a value of 0. In stimulation assays, the unstimulated value was subtracted from the stimulated to the determine absolute change in biomarker level.

### Data Analysis

Baseline characteristics were summarized using proportions or median values with interquartile ranges (IQRs). Characteristics between survivors and nonsurvivors were compared using the Wilcoxon rank sum test, *t* test, or Fisher exact test, as appropriate. Data from flow cytometry and biomarker analysis on unstimulated and stimulated blood were combined and analyzed using principal component analysis (PCA) and unsupervised hierarchical cluster analysis as described elsewhere [16]. Before PCA, skewed variables were log_2_ transformed and scaled, such that the geometric mean equaled 0 and variance equaled 1. Variables were filtered using statistical tests prior to incorporation into PCA plots and cluster analysis, such that only variables with a statistically significant association with the dependent variable (14-day mortality) were used. Statistical significance was defined as a *P* value of <.05 and a *Q* value of <.1 (equivalent to a 10% false-discovery rate [FDR], using the Benjamini–Hochberg procedure for multiple-testing correction [17]). For initial PCA, missing values were imputed using the K nearest-neighbors technique [18]; subsequent logistic regression analysis was restricted to 37 participants who had all 205 baseline variables measured. Analysis was performed using Stata, version 12. (Stata, College Station, Texas), and Qlucore Omics Explorer, version 3.0 (Qlucore, Lund, Sweden).

## RESULTS

### Participants

Sixty participants were enrolled, with a median age of 36 years (IQR, 30–43 years) and median CD4^+^ T-cell count of 34 cells/µL (IQR, 13–76 cells/µL); 17 (28%) were taking ART at enrollment. Mortality was 23% at 2 weeks (14 of 60 patients) and 39% at 12 weeks (22 of 57). Three participants were lost to follow-up after hospital discharge. Of 14 deaths within 2 weeks, attributable causes of death were cryptococcal meningitis (n = 10), pulmonary cryptococcosis (n = 1), pneumonia (n = 1), and multiorgan failure of unknown etiology (n = 2). Of 8 deaths between 2 and 12 weeks, attributable causes of death were cryptococcal meningitis (n = 1), cryptococcal-related immune reconstitution inflammatory syndrome (n = 1), *Klebsiella pneumoniae* bacteremia (n = 1), ruptured iliac aneurysm (n = 1), and unknown (n = 4). Blood cultures were performed at study enrollment for 46 participants (35 survivors and 11 nonsurvivors); *C. neoformans* grew in 20 (43.5%). No episodes of bacteremia were identified in any participant at enrollment, but 32 had received ceftriaxone on hospital admission. Microbiologically confirmed tuberculosis was diagnosed in 9 patients within 2 months of study enrollment. No study deaths were attributable to tuberculosis.

### Clinical and Laboratory Differences Between Subjects Who Died and Those Who Survived

Persons who died by day 14 were significantly more likely than survivors to have impaired consciousness at presentation (43% vs 11%; *P* = .014). Nonsurvivors were also more likely to have evidence of gaze palsy (29% vs 7%; *P* = .045), higher CSF quantitative culture (median, 5.5 log_10_ CFU/mL [IQR, 4.7–6.4 log_10_ CFU/mL] vs 4.5 log_10_ CFU/mL [IQR, 3.1–5.5 log_10_ CFU/mL]; *P* = .032), higher circulating WBC counts (median, 6.5×10^9^ cells/L [IQR, 3.9×10^9^–7.4×10^9^ cells/L] vs 4.4×10^9^ cells/L [IQR, 2.8×10^9^–5.8×10^9^ cells/L]; *P* = .020), and higher serum C-reactive protein levels (median, 85 mg/L [IQR, 46–115 mg/L] vs 33 mg/L [IQR, 13–68 mg/L]; *P* = .011). There was no significant difference observed in baseline CD4^+^ T-cell count, HIV load, or ART status between survivors and nonsurvivors (Table 1).

**Table 1.** Differences in Baseline Characteristics Between Participants Who Died and Survived by Day 14

Variable	Survived to Day 14 (n = 46)	Died by Day 14 (n = 14)	*P* Value^a^
Age, y	36 (30–43)	37 (27–45)	.854
Male sex	25 (54)	8 (57)	.999
CD4^+^ T-cell count, cells/µL	35 (12–85)	33 (13–45)	.546
HIV load, log_10_ copies/mL	5.1 (4.4–5.41)	5.2 (4.7–5.7)	.318
Receiving ART	14 (30)	3 (21)	.737
Active tuberculosis^b^	7 (15)	2 (14)	.999
Altered consciousness	5 (11)	6 (43)	.014
Seizures	8 (17)	3 (21)	.707
Gaze palsy	3 (7)	4 (29)	.045
CSF culture, log_10_ CFU/mL	4.5 (3.1–5.5)	5.5 (4.7–6.4)	.032
CSF OP at enrollment, cm H_2_O	27 (19–36)	22 (14–41)	.577
Maximum CSF OP, cm H_2_O	36 (27–47)	31 (16–49.5)	.308
CSF WBC count, cells/µL	19.5 (3–106)	5.5 (1–45)	.258
CSF protein level, g/L	1.04 (0.65–1.52)	0.69 (0.46–1.43)	.311
CSF glucose level, mmol/L	2.1 (1.6–2.7)	3.0 (2.1–4.4)	.015
Blood WBC count, ×10^9^ cells/L	4.4 (2.83–5.79)	6.46 (3.86–7.36)	.020
CRP level, mg/L	33 (13–68.3)	85 (46.5–115)	.011

Data are medians with IQR, or numbers with percentages.

Abbreviations: ART, antiretroviral therapy; CFU, colony-forming units; CRP, C-reactive protein; CSF, cerebrospinal fluid; IQR, interquartile range; OP, opening pressure; WBC, white blood cell.

^a^ By the Wilcoxon rank sum or Fisher exact tests, as appropriate.

^b^ Defined as microbiologically confirmed tuberculosis ± 2 months of study enrollment

### Differences in Blood Immune Response Between Subjects Who Died and Those Who Survived

**Figure 2.**
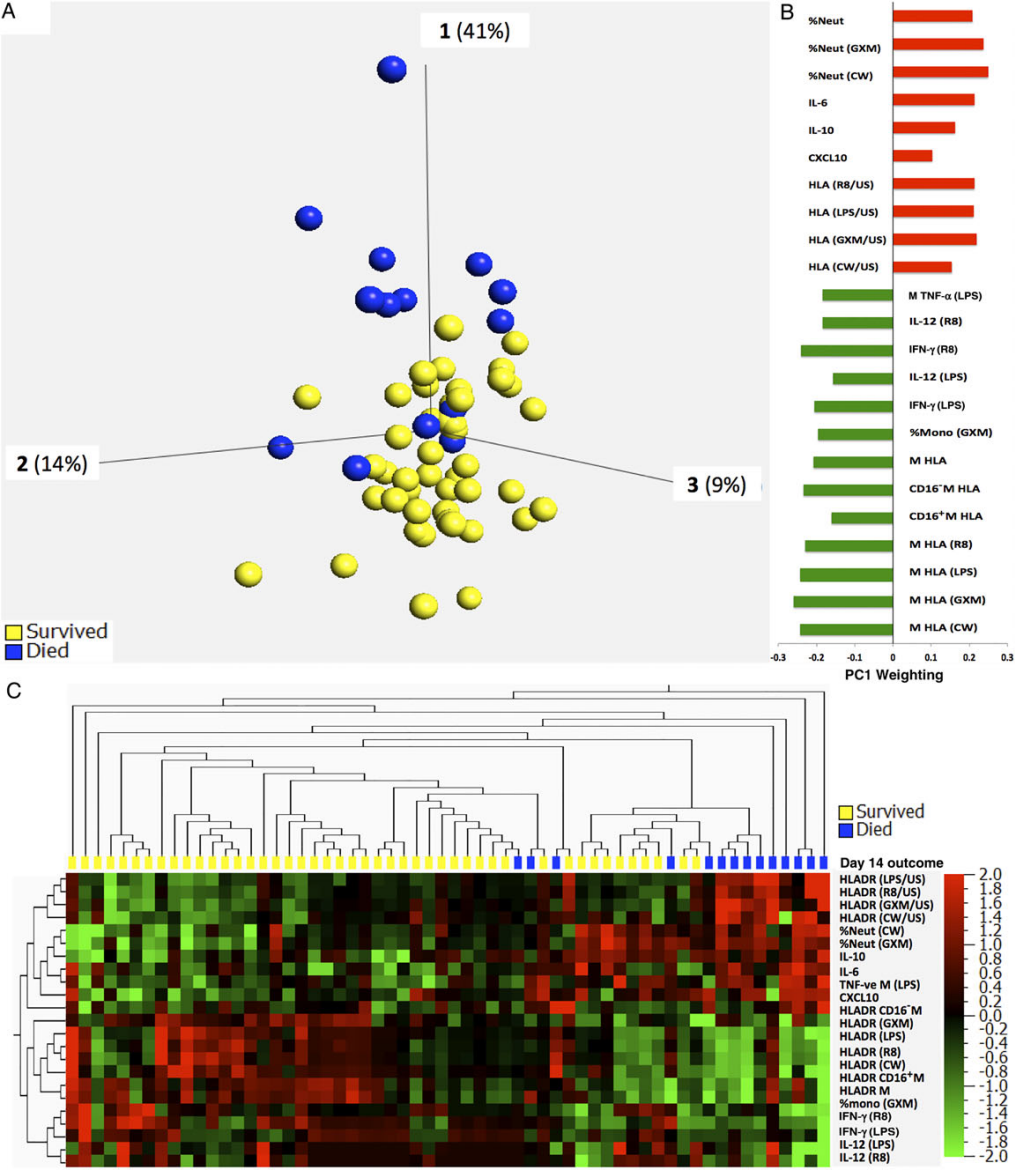
Principal component analysis (PCA) and unsupervised hierarchical clustering illustrating differences in baseline immune response between subjects who died or survived by day 14. *A*, PCA shows distribution of subjects according to baseline blood immune response. Study subjects are represented by dots, colored by outcome. The 3 axes shown refer to the first 3 principal components; their contribution to the total sample variation is shown as a percentage. Subjects who died (blue) cluster together and are largely separated from those who survived (yellow), owing to higher values for principal component 1 (PC1). *B*, Variables that contributed to PC1 and their respective weighting (green bars, negative weighting; red bars, positive weighting). Stimulating agents or the ratio between stimulated and unstimulated (US) are listed in brackets. *C*, Heat map illustrating unsupervised hierarchical clustering of participants (columns) according to the 23 variables contributing to PC1 (rows; green square, variable decreased; red square, variable increased). Subjects clustered by day 14 outcome (blue, died; yellow, survived). Abbreviations: CD16^+^M, CD14^++^CD16^+^ intermediate monocytes; CD16^−^M, CD14^++^CD16^−^ classical monocytes; CW, heat-killed, mechanically disrupted *Cryptococcus*; GXM, glucuronoxylomannan; HLA, HLA-DR expression (mean fluorescence intensity); IFN-γ, interferon γ; IL-6, interleukin 6; IL-10, interleukin 10; IL-12, interleukin 12; LPS, lipopolysaccharide; M, monocytes; Neut, neutrophils; R8, R848 (TLR7/8 agonist); TNF-α, tumor necrosis factor α; US, unstimulated.

A clear difference in peripheral blood immune response was noted between nonsurvivors and survivors. PCA showed distinct clustering of participants according to day 14 outcome, with persons who died having significantly higher values for principal component 1 (PC1; Figure 2*A*). Twenty-three variables contributed to PC1, and levels of all differed significantly between survivors and nonsurvivors (*P* < .05 and *Q* < 0.1), resulting in a distinct immune signature (Figure 2*B* and Table 2). This was characterized by an increased proportion of circulating neutrophils; higher serum concentrations of IL-6, IL-10, and CXCL10; decreased expression of HLA-DR on circulating monocytes (both resting and stimulated with LPS or R848); a decreased proportion of monocytes producing TNF-α when stimulated with LPS; and reduced concentrations of IL-12 and IFN-γ when whole blood was stimulated with LPS or R848 (Table 2). Although HLA-DR ratios (stimulated/unstimulated) were significantly higher in nonsurvivors, this was due to low expression by unstimulated cells. Unsupervised hierarchical clustering using the same 23 variables also demonstrated clear grouping of participants by day 14 outcome (Figure 2*C*). A similar immune signature was identified in participants who died by week 12, but this was not significant when adjusted for multiple comparisons (data not shown).

**Table 2.** Differences in Variables Contributing to Principal Component 1 (PC1) Between Subjects Who Died or Survived by Day 14

Variable	Stimulation	Survived		Died		*P* Value^a^	*Q*^b^
		n	Median (IQR)^c^	n	Median (IQR)^c^		
Cell frequencies, % of WBCs							
Neutrophils	None	46	67.5 (54.6–75.8)	14	76.8 (68.8–82.6)	.006	0.088
Neutrophils	GXM	31	54.2 (39.6–65.5)	11	70.8 (68.5–73.6)	.003	0.058
Neutrophils	CW	30	54.1 (44.9–70.8)	11	75.0 (65.1–88.7)	.003	0.058
Monocytes	GXM	31	4.86 (3.14–5.76)	11	2.98 (0.91–4.35)	.006	0.093
Serum cytokines, pg/mL							
IL-6	None	46	27 (13–57)	14	85 (18–170)	.009	0.097
IL-10	None	46	5.6 (1.5–11.3)	14	16.3 (10.9–35.2)	.001	0.030
CXCL10	None	46	76 (58–126)	14	127 (79–272)	.009	0.097
Monocyte activation^d^							
HLA-DR	None	43	1373 (807–2073)	13	865 (367–995)	.009	0.097
HLA-DR CD16^−^	None	43	1113 (675–1591)	13	518 (351–896)	.002	0.057
HLA-DR CD16^+^	None	43	6723 (5357–9860)	13	3491 (2623–6966)	.003	0.058
HLA-DR	R8	32	5880 (4027–8257)	13	3495 (1775–5694)	.009	0.097
HLA-DR	LPS	33	5648 (4356–7730)	13	3173 (2058–5881)	.003	0.058
HLA-DR	GXM	31	4360 (3754–6179)	11	2033 (1352–5254)	.001	0.028
HLA-DR	CW	30	3181 (2367–3974)	11	1115 (551–3470)	.002	0.057
HLA-DR ratio	R8/US	32	1.85 (1.50–2.25)	13	2.95 (2.47–4.51)	<.001	<0.001
HLA-DR ratio	LPS/US	33	1.75 (1.47–2.02)	13	3.59 (2.12–4.81)	<.001	<0.001
HLA-DR ratio	GXM/US	31	1.35 (1.16–1.56)	11	2.47 (1.64–3.34)	<.001	<0.001
HLA-DR ratio	CW/US	30	0.94 (0.67–1.16)	11	1.59 (1.19–2.21)	.001	0.030
TNF positivity–%	LPS	35	48 (37–58)	13	13 (10–41)	.003	0.058
Whole-blood responses, pg/mL^e^							
IFN-γ	R848	33	85 (35–469)	13	43 (31–67)	.011	0.099
IL-12	R848	33	7829 (4802–12571)	13	4168 (2951–9368)	.010	0.099
IFN-γ	LPS	33	32.3 (30.5–43.4)	13	30.5 (30.0–37.1)	.009	0.097
IL-12	LPS	33	2783 (1953–3975)	13	2204 (1112–2867)	.009	0.097

Abbreviations: CD16^+^, CD14^++^CD16^+^ (intermediate monocytes); CD16^−^, CD14^++^CD16^−^, classical monocytes; CW, heat-killed, mechanically disrupted *C. neoformans* H99; GXM, glucuronoxylomannan; IFN-γ, interferon γ; IL-6, interleukin 6; IL-10, interleukin 10; IL-12, interleukin 12; LPS, lipopolysaccharide (Toll-like receptor 4 agonist); R8, R848 (Toll-like receptor 7/8 agonist); TNF, tumor necrosis factor; US, unstimulated; WBC, white blood cell.

^a^ Calculated using independent *t* tests.

^b^ Calculated using the Benjamini–Hochberg principal for multiple comparisons as previously described [18].

^c^ Medians and interquartile ranges (IQRs) are displayed for clarity, but statistical testing was performed using parametric tests with log-transformed variables normalized to the geometric mean.

^d^ Data are median fluorescence intensity, unless indicated.

^e^ Data refer to absolute difference in relation to unstimulated sample.

### Predictive Ability of Blood Immune Signature

To further explore the association between this immune signature and poor outcome, the first principal component from the analysis (PC1) was added as a summative variable to a logistic regression model predicting death by day 14. Analysis was restricted to 37 individuals for whom full immunological data were available (28 survivors and 9 nonsurvivors). PC1 was found to be significantly associated with day 14 mortality both on univariate analysis and multivariate analysis adjusted for GCS and CSF fungal burden (unadjusted odds ratio [OR], 1.9 [95% confidence interval {CI}, 1.2–3.1; *P* = .007]; adjusted OR, 2.02 [95% CI, 1.2–3.4; *P* < .001]; Table 3). Adding PC1 to a base model consisting of altered consciousness level and CSF fungal burden resulted in significantly improved model fit (likelihood ratio test *P* = .0001). To ensure that these findings were applicable to the rest of the cohort, PC1 was recalculated without the 16 variables derived from whole-blood antigen-stimulation assays (the main reason for missing data). The 7 remaining variables comprised HLA-DR expression on classical monocytes, intermediate monocytes, and monocytes as a whole; neutrophil frequency (percentage of WBCs); and serum concentrations of IL-6, IL-10, and CXCL10. Data were available for 55 subjects in the cohort (42 survivors and 13 nonsurvivors). Despite the removal of 16 variables, limited PC1 remained significantly associated with day 14 mortality on both univariate and multivariate analyses (unadjusted OR, 2.55 [95% CI, 1.4–4.5; *P* = .002]; adjusted OR, 2.89 [95% CI, 1.5–5.8; *P* < .001]; Table 3). The addition of limited PC1 also significantly improved the fit of the model, compared with BASE model (likelihood ratio test *P* < .0001). In both the PC1 and the limited PC1 model, adjusting the model for CD4^+^ T-cell count, ART status, and amphotericin B status at enrollment or excluding the 3 noncryptococcal deaths did not significantly alter these findings (Supplementary Tables 1 and 2). Adding PC1 or limited PC1 to the base model also resulted in increased area under the curve on receiver operating characteristic curve analysis, but this did not reach statistical significance (*P* = .107 and .074, respectively; Supplementary Figure 1).

**Table 3.** Logistic Regression Models Illustrating the Association Between Immune Signature (PC1) and Day 14 Mortality, Adjusting for Altered Consciousness and Fungal Burden

Variable	Adjusted OR (95% CI)	*P* Value
Full PC1 model (n = 37)^a^		
Altered consciousness	6.39 (.45–90)	.152
CSF quantitative culture, log_10_ CFU/mL	2.09^b^ (.65–6.7)	.141
PC1 (full)	2.02^b^ (1.19–3.43)	<.001
Limited PC1 model (n = 55)^c^		
Altered consciousness	20.6 (1.35–314)	.012
CSF quantitative culture, log_10_ CFU/mL	2.37^b^ (.89–6.3)	.034
PC1 (limited)	2.89^b^ (1.45–5.75)	<.001

Abbreviations: CI, confidence interval; CFU, colony-forming units; CSF, cerebrospinal fluid; GCS, Glasgow coma scale; OR, odds ratio; PC, principal component.

^a^ Full PC1 calculated for 37 subjects, using 23 variables.

^b^ OR expressed as per unit increase.

^c^ Limited PC1 calculated for 55 subjects, using 7 variables: monocyte HLA-DR expression (classical, intermediate, and entire population), proportion of neutrophils, and serum concentrations of interleukin 6, interleukin 10, and CXCL10.

### Reasons for an Antiinflammatory Response in Nonsurvivors

**Figure 3.**
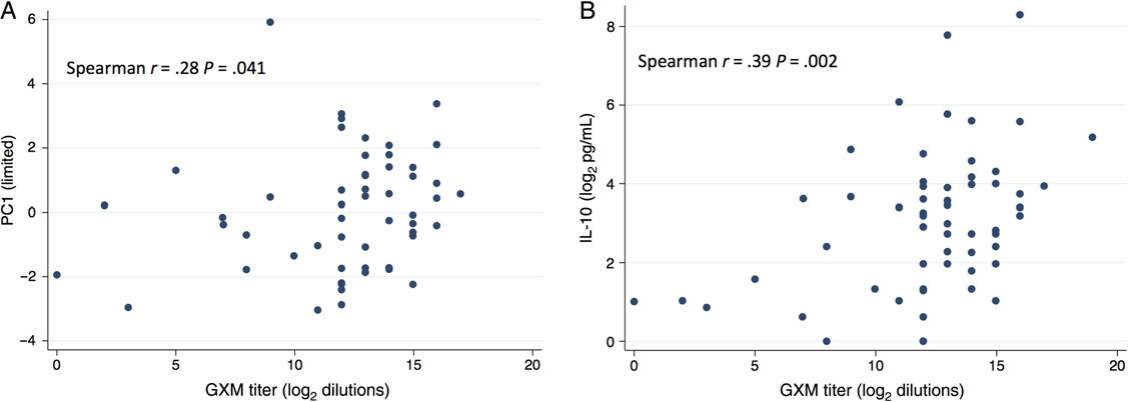
Relationship between cryptococcal antigen load and blood immune response. *A*, Scatterplot demonstrating positive correlation between glucuronoxylomannan (GXM) titer and values for limited principal component 1 (PC1). *B*, Scatterplot demonstrating significant positive correlation between serum GXM titer and interleukin 10 concentration.

We hypothesized that the immune phenotype observed in nonsurvivors was a compensatory downregulation of cellular responses following a period of immune activation. Possible causes of this immune activation included disseminated cryptococcal infection*,* HIV infection, or occult bacterial sepsis. To explore the first 2 possibilities, associations between PC1 scores and parameters approximating these factors were examined. A statistically significant positive correlation was noted between limited PC1 scores and serum GXM titers (Spearman r = 0.28; *P* = .041; Figure 3*A*) but not with plasma HIV load (Spearman rho = 0.08; *P* = .561). When the relationship between GXM and individual components of limited PC1 were examined, a positive correlation was noted with the percentage of neutrophils in blood (Spearman rho = 0.33; *P* = .010), serum CXCL10 concentrations (Spearman rho = 0.31; *P* = .015), and serum IL-10 concentrations (Spearman rho = 0.39; *P* = .002; Figure 3*B*).

## DISCUSSION

In this prospective cohort study, we examined clinical and immune correlates of fatal outcome in people with HIV-associated cryptococcal meningitis. Similar to other cohorts, nonsurvivors were significantly more likely to have a reduced consciousness level at presentation, a higher CSF fungal burden, and increased numbers of circulating blood leukocytes [8]. PCA and unsupervised hierarchical clustering analysis demonstrated clear differences in the baseline blood immune response between subjects who survived or died by day 14, with nonsurvivors having significantly higher scores for PC1. A breakdown of the variables contributing to PC1 revealed an immune signature associated with mortality, characterized by a higher proportion of circulating neutrophils, increased levels of soluble markers of immune activation (IL-6 and CXCL10), higher concentrations of antiinflammatory mediators (IL-10), reduced Th1 cytokine (IL-12 and IFN-γ) production in supernatant following incubation of whole blood with TLR4 or TLR7/8 agonists, decreased HLA-DR expression on circulating monocytes (both stimulated and unstimulated), and decreased monocyte production of TNF-α following LPS stimulation. This immune signature remained an independent determinant of 14-day mortality even when adjusted for baseline fungal burden, consciousness level, CD4^+^ T-cell count, ART status, and amphotericin B status, and even if noncryptococcal deaths were excluded.

The combination of decreased monocyte expression of HLA-DR and reduced monocyte TNF-α response to LPS has been referred to by some authors as “monocyte anergy” [19–21]. It has previously been described in bacterial sepsis, acute pancreatitis, and severe trauma, in which it is frequently associated with a worse clinical outcome, such as increased frequency of nosocomial infections and increased mortality [19, 22–25]. Monocyte anergy is one component of a more widespread downregulation of the immune response known as the compensatory antiinflammatory response syndrome (CARS), which typically occurs following a severe proinflammatory insult. Additional features of CARS include increased lymphocyte apoptosis, cutaneous anergy, reduced T-cell proliferation and proinflammatory cytokine (IL-2 and TNF-α) responses, and increased concentrations of IL-10 [21, 25–27]. Increased plasma IL-10 levels were noted among nonsurvivors in our study and may explain the observed alterations in ex vivo cellular immune responses. In vitro studies have shown that IL-10 directly inhibits proinflammatory cytokine production and antigen presentation by macrophages, resulting in additional impairment of CD4^+^ T-cell production of IFN-γ [28, 29]. Murine studies have also shown that significantly impaired T-cell proliferation and decreased IFN-γ production occur when monocyte anergy is induced through preexposure to LPS, an IL-10–dependent phenomenon known as endotoxin tolerance [30–32]. Although T-cell responses were not examined specifically in this study, subjects who died by day 14 had decreased production of IL-12 and IFN-γ when stimulated with TLR4 or TLR7/8 agonists in whole-blood assays, suggesting a decreased ability to mount a Th1 response. IFN-γ is widely recognized to be a central component of the protective Th1 host response against *C. neoformans*. Low CSF concentrations of IFN-γ and a blood CD4^+^ T-cell phenotype characterized by a lack of IFN-γ and TNF-α production have been observed in HIV-infected individuals who died from cryptococcal meningitis [8, 12]. Furthermore, IFN-γ therapy has been shown to increase the rate of fungal clearance when added to amphotericin B and flucytosine in a clinical trial [13]. IL-12 is produced by monocytes, macrophages, and dendritic cells and acts during antigen presentation to promote IFN-γ production by natural killer and CD4 T cells [30, 33–35]. The low levels of IL-12 production following whole-blood stimulation along with decreased expression of the major histocompatibility complex class II molecule HLA-DR on deactivated (or “anergic”) monocytes observed in this study suggest that impaired antigen presentation may be an explanation for the impaired Th1 responses previously observed in nonsurvivors. In addition to impairing anticryptococcal responses, such an immune phenotype may also predispose to further infections with bacterial pathogens, as observed in patients with severe sepsis [27].

There are a number of possible explanations why a CARS-like immune response was observed among HIV-infected individuals who died from cryptococcosis. The significant correlations between serum titers of GXM (the major component of the cryptococcal capsule), PC1, and serum IL-10 provide supportive evidence that this immune phenotype may have been driven by the cryptococcal organism and its antigens. This hypothesis is further supported by in vitro studies showing that GXM provokes a proinflammatory cytokine response in neutrophils (IL-1β, IL-6, IL-8, and TNF-α) but leads to an antiinflammatory response in monocytes (production of IL-10 and impaired antigen presentation) [36–38]. Given the massive tissue burden of cryptococci observed in HIV-associated cryptococcosis [39], it is plausible that the immune phenotype observed among nonsurvivors in this study was a direct effect of GXM, possibly mediated by the actions of IL-10. This is supported by in vitro work demonstrating that the addition of GXM to monocytes in cell culture results in impaired antigen presentation and reduced T-cell responses via an IL-10–dependent mechanism [38].

An alternative explanation is that the immune signature represents immune exhaustion due to persistent HIV-associated immune activation, an increasingly recognized component of HIV pathogenesis [40, 41]. IL-10 levels are known to increase as HIV infection progresses, and high levels of serum IL-6 (along with C-reactive protein) have been repeatedly associated with fatal outcome [42–45]. Possible mechanisms for this include translocation of bacterial products through the intestinal wall [46, 47] or direct effects of HIV on the host immune response, possibly through direct stimulation of monocytes by HIV proteins [48]. However, there was no significant association noted between PC1 and HIV load, and sCD14—a marker of monocyte activation following stimulation with LPS and frequently used as a surrogate marker of gut translocation [47]—did not form part of the immune signature identified among nonsurvivors.

Finally, the observed immune phenotype could conceivably be due to coexistent but clinically unrecognized bacterial infection. Although no blood cultures performed at study enrollment identified any bacterial pathogens, the widespread use of ceftriaxone on admission to the hospital will have significantly reduced the sensitivity of this.

There are a number of limitations to this study. It was exploratory in nature with a relatively small sample size and thus was underpowered for certain analyses. As the cohort was not split into training and testing sets, the predictive ability of this immune signature remains unvalidated. It is therefore important to repeat these observations in independent cohorts to assess reproducibility. The study also suffered from a degree of missing data, as an ambitious number of immune assays were performed, the majority of them in real time on fresh samples of peripheral blood. However, this did not appear to alter the final results. The immune signature remained significantly associated with 14-day mortality either when restricted to the 37 persons with a full set of observations, when a limited signature was applied to 55 persons, or when an accepted method of imputing missing data was used for the whole data set.

Despite these limitations, this study presents new insights regarding the systemic immune dysfunction associated with death in HIV-associated cryptococcal meningitis. A clear immune signature was identified at baseline in the blood of nonsurvivors, characterized by increased inflammation, monocyte deactivation (or anergy), and downregulation of Th1 responses. This immune signature remained an independent determinant of mortality even after adjustment for other baseline factors. Given the known immune modulating effects of GXM and the strong association between serum GXM titer and IL-10 concentration observed in this cohort, it is plausible that this may be a direct consequence of disseminated infection with *C. neoformans*. Further work is required to define the mechanism and develop strategies to reverse the immune phenomenon.

## Supplementary Data

Supplementary materials are available at http://jid.oxfordjournals.org. Consisting of data provided by the author to benefit the reader, the posted materials are not copyedited and are the sole responsibility of the author, so questions or comments should be addressed to the author.

Supplementary Data
