# Supplementary material for: A Glucuronoxylomannan-Associated Immune Signature, Characterized by Monocyte Deactivation and an Increased Interleukin 10 Level, Is a Predictor of Death in Cryptococcal Meningitis
Source: J Infect Dis. 2016 Jan 14;213(11):1725–34. doi: 10.1093/infdis/jiw007 (PMC4857465; doi:10.1093/infdis/jiw007)
Supplement: Supplementary Data [file supp_213_11_1725__index.html]

A Glucuronoxylomannan-Associated Immune Signature, Characterized by Monocyte Deactivation and an Increased Interleukin 10 Level, Is a Predictor of Death in Cryptococcal Meningitis — A Glucuronoxylomannan-Associated Immune Signature, Characterized by Monocyte Deactivation and an Increased Interleukin 10 Level, Is a Predictor of Death in Cryptococcal Meningitis — Supplementary Data 

# A Glucuronoxylomannan-Associated Immune Signature, Characterized by Monocyte Deactivation and an Increased Interleukin 10 Level, Is a Predictor of Death in Cryptococcal Meningitis

## Supplementary Data

Supplementary Data

- Supplementary Data - Docx file
- Supplementary Table 1 - docx file
- Supplementary Figure 1 - pdf file
- Supplementary Table 2 - docx file
