# Supplementary material for: A Glucuronoxylomannan-Associated Immune Signature, Characterized by Monocyte Deactivation and an Increased Interleukin 10 Level, Is a Predictor of Death in Cryptococcal Meningitis
Source: J Infect Dis. 2016 Jan 14;213(11):1725–34. doi: 10.1093/infdis/jiw007 (PMC4857465; doi:10.1093/infdis/jiw007)
Supplement: Supplementary Data [file supp_jiw007_jiw007supp.docx]

## Supplementary Methods

### Whole blood flow cytometry

Panel 1: CD3-PacBlue, CD16-APCH7 (BD Biosciences); CD8-Qdot655, CD14-Qdot605 (Invitrogen); CD4-PECy7, HLADR-AF700, CD163-APC, CD38-PE, PD1-PerCPCy5.5 and CD19-AF488 (Biolegend). Panel 2: CD14-Qdot605 (Invitrogen); CD16-APCH7 (BD Biosciences); CCR2-AF488 (R&D); HLADR-AF700, CD163-APC, CCR5-PacBlue, CD11c-PerCPCy5.5, TLR4-PE and CD80-PECy7 (Biolegend). Cells were analyzed on a BD LSR Fortessa Flow Cytometer configured daily with CS&T beads (BD Biosciences); 150,000 total events were recorded. Species appropriate compensation beads were used to ensure accurate compensation (BD Biosciences). Acquisition and analysis of data was performed using FACS Diva software (BD Biosciences, San Jose, CA, USA) and FlowJo version 9.5.3 (Tree Star software, OR, USA) respectively. Fluorescence minus one was used to ensure accurate gating [Herzenberg LA, et al. Nat Immunol **2006**; 7:681–685].

**Preparation of glucuronoxylomannan (GXM)**

GXM was obtained from liquid culture supernatants of *C. neoformans* using ultrafiltration as described elsewhere [Nimrichter L., et al. Eukaryotic Cell **2007**; 6:1400–1410]. The strain used was KN99-α [Nielsen, K., et al. *Infection and Immunity* **2003**; *71*(9), 4831–4841]. Cryptococcal preparations were tested using a *Limulus* Amebocyte Lysate assay to ensure they were free of endotoxin contamination (Associates of Cape Cod Inc., MA, USA).

### Antigen stimulation assay

Cells were stained using CD14-Qdot605 (Invitrogen), HLADR-AF700, CD66a/c/e-AF488, CD19-AF488, CD3-AF488 (Biolegend). After erythrocyte lysis and permeabilization, intracellular cytokine staining was performed with IL6-APC, TNFα-PECy7, IL10-PE, and IL12-PacBlue (Biolegend). Cells were analyzed on a BD LSR Fortessa as described above; 500,000 total events were recorded. Unstimulated samples were used for gating.
