# Supplementary material for: A Glucuronoxylomannan-Associated Immune Signature, Characterized by Monocyte Deactivation and an Increased Interleukin 10 Level, Is a Predictor of Death in Cryptococcal Meningitis
Source: J Infect Dis. 2016 Jan 14;213(11):1725–34. doi: 10.1093/infdis/jiw007 (PMC4857465; doi:10.1093/infdis/jiw007)
Supplement: Supplementary Data [file supp_jiw007_jiw007supp_table1.docx]

**Supplementary Table 1. Multivariate analysis demonstrating effect of peripheral blood immune signature on day 14 mortality adjusting for altered consciousness, fungal burden, Amphotericin B treatment, ART status and CD4 count.**

|  | Adjusted Odds Ratio | *P*-value | 95%CI |
| --- | --- | --- | --- |
| Full PC1 model (n=37)^a^ |  |  |  |
| PC1 (full) | 2.1* | 0.009 | 1.2-3.6 |
| Altered consciousness | 4.1 | 0.332 | 0.24-69 |
| CSF quantitative culture, log_10_CFU/mL | 2.3* | 0.151 | 0.7-7.5 |
| CD4, log_2_ cells/μL | 1.4* | 0.385 | 0.7-3 |
| Taking ART at enrolment | 1.3 | 0.886 | 0.1-17 |
| Taking Amphotericin B at enrolment | 1.4 | 0.806 | 0.1-20 |
| Limited PC1 model (n=55)^b^ |  |  |  |
| PC1 (limited) | 2.9* | 0.002 | 1.5-5.8 |
| Altered consciousness | 24.8 | 0.033 | 1.3-477 |
| CSF quantitative culture, log_10_CFU/mL | 2.4* | 0.119 | 0.8-7.5 |
| CD4, log_2_ cells/μL | 1.1* | 0.657 | 0.6-2.0 |
| Taking ART at enrolment | 0.5 | 0.557 | 0.05-5.1 |
| Taking Amphotericin B at enrolment | 0.3 | 0.319 | 0.03-3.1 |

**^a^**Variables influencing PC1 (full) are detailed in Figure 2b and Table 2.

**^b^**Variables contributing to PC1 (limited) include HLA-DR expression on monocytes (classical, intermediate and entire population), proportion of circulating neutrophils, and serum concentrations of IL-6, IL-10, and CXCL10.*Odds Ratio is per unit increase

Abbreviations: OR = Odds ratio; PC = principal component; CSF = Cerebrospinal fluid; CFU = colony forming units; ART = anti-retroviral therapy
