# Supplementary material for: A Glucuronoxylomannan-Associated Immune Signature, Characterized by Monocyte Deactivation and an Increased Interleukin 10 Level, Is a Predictor of Death in Cryptococcal Meningitis
Source: J Infect Dis. 2016 Jan 14;213(11):1725–34. doi: 10.1093/infdis/jiw007 (PMC4857465; doi:10.1093/infdis/jiw007)

Supplementary Figure 1. Receiver Operator Characteristic curves illustrating the effect of adding PC1 to a base model in predicting 14-day mortality. (a) Full PC1 model incorporating 23 variables identified in Figure 2b Table 2. **(b)** Limited PC1 model incorporating seven variables detailed in Table 3. BASE model constructed using altered consciousness and fungal burden. Addition of PC1 in (a) and limited PC1 in (b) resulted in increased area under the curve but did not reach statistical significance.

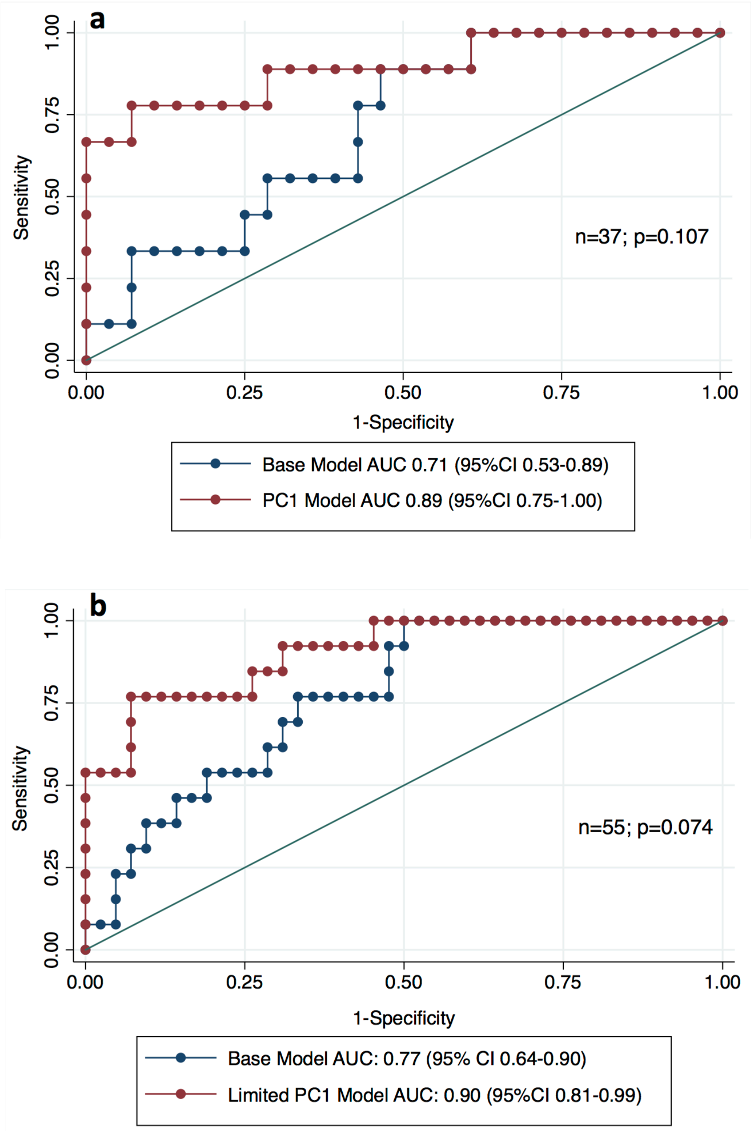

Supplement: Supplementary Data [file supp_jiw007_jiw007supp_fig1.pdf]
