# Supplementary material for: A Glucuronoxylomannan-Associated Immune Signature, Characterized by Monocyte Deactivation and an Increased Interleukin 10 Level, Is a Predictor of Death in Cryptococcal Meningitis
Source: J Infect Dis. 2016 Jan 14;213(11):1725–34. doi: 10.1093/infdis/jiw007 (PMC4857465; doi:10.1093/infdis/jiw007)
Supplement: Supplementary Data [file supp_jiw007_jiw007supp_table2.docx]

**Supplementary Table 2. Multivariate analysis demonstrating effect of peripheral blood immune signature on Day-14 cryptococcal mortality^a^**

|  | Adjusted Odds Ratio | *P*-value | 95%CI |
| --- | --- | --- | --- |
| Full PC1 model (n=35)^b^ |  |  |  |
| Altered consciousness | 8.3 | 0.131 | 0.5-145 |
| CSF quantitative culture, log_10_CFU/mL | 1.9* | 0.273 | 0.5-7.3 |
| PC1 (full) | 2.1* | 0.001 | 1.2-3.9 |
| Limited PC1 model (n=52)^c^ |  |  |  |
| Altered consciousness | 27.3 | 0.008 | 1.4-520 |
| CSF quantitative culture, log_10_CFU/mL | 2.56* | 0.040 | 0.8-7.9 |
| PC1 (limited) | 2.78* | 0.001 | 1.3-5.9 |

^a^Excluding three participants whose deaths could not be clearly attributable to cryptococcosis

**^b^**Variables influencing PC1 (full) are detailed in Figure 2b and Table 2.

**^c^**Variables contributing to PC1 (limited) include HLA-DR expression on monocytes (classical, intermediate and entire population), proportion of circulating neutrophils, and serum concentrations of IL-6, IL-10, and CXCL10.*Odds Ratio is per unit increase

Abbreviations: PC = principal component; CSF = Cerebrospinal fluid; CFU = colony forming units; ART = anti-retroviral therapy
